# Preparation of Bioactive Polyamide Fibres Modified with Acetanilide and Copper Sulphate

**DOI:** 10.3390/ma16206789

**Published:** 2023-10-20

**Authors:** Dorota Biniaś, Włodzimierz Biniaś, Czesław Ślusarczyk, Alicja Machnicka

**Affiliations:** 1 Department of Environmental Protection and Engineering, Faculty of Materials, Civil and Environmental Engineering, University of Bielsko-Biala, ul. Willowa 2, 43-309 Bielsko-Biala, Poland; amachnicka@ath.bielsko.pl; 2 Department of Materials Science, Faculty of Materials, Civil and Environmental Engineering, University of Bielsko-Biala, ul. Willowa 2, 43-309 Bielsko-Biala, Poland; wbinias@ath.bielsko.pl (W.B.); cslusarczyk@ath.bielsko.pl (C.Ś.)

**Keywords:** PA6, fibres, additives, acetanilide, copper sulphate, structural analysis, antibacterial activity

## Abstract

This paper presents a simple method of obtaining polyamide 6 fibres modified with acetanilide and copper ions. During the spinning of the fibres with the additives applied, a partial reduction of CuSO_4_ to Cu^2+^ and Cu^+^ ions occurs, which is observed as a change in the blue colour of the prepared polyamide granulate to the grey–brown colour of the formed fibres. CuMPs obtained as a result of the salt reduction should give the obtained fibres bioactive properties. Three types of microorganisms were selected to assess the microbiological activity of the obtained fibres, i.e., Gram-positive *Staphylococcus aureus* and Gram-negative *Pseudomonas aeruginosa* and *Escherichia coli*. The fibres have antibacterial activity against Gram-positive and Gram-negative bacteria. The largest inhibition zones were obtained for the Gram-positive bacteria *Staphylococcus aureus*, ranging from 1.5 to 4.5 mm, depending on the concentration of CuMPs. The morphology of the fibres’ surfaces was examined by means of scanning electron microscopy (SEM) and optical microscopy (OM). The changes in the polymer structure chemistry are studied by Fourier transform infrared spectroscopy (FTIR), Raman spectroscopy, X-ray structure studies (WAXS and SAXS) and an energy-dispersive spectroscopy (EDS) analysis. The newly obtained bioactive polyamide fibres can be used in many areas, including medicine, clothing and environmental protection for the production of filters.

## 1. Introduction

Polyamides (PAs) are a wide group of polymers used in many industries, including the production of components used in the automotive industry, construction, environmental protection and medical applications [1,2,3,4,5,6]. Compared to other polymers, polyamides are characterized by relatively superior mechanical properties, resistance to stretching and abrasion and a low coefficient of friction.

The processing temperature varies depending on the type of polyamide that contains an olefin fragment in the polymer [7,8,9]. Polyamides can be formed through standard processing methods used for thermoplastic polymers, i.e., by extrusion, in which fibres, foils or semi-finished products for the production of structural elements are obtained using injection, thermoforming or 3D printing.

As a result of dissolving PAs in appropriate solvents and using the electrospinning method to form fibres from the solution, we can obtain micro- and nanopolyamide fibres for special applications, which can be used in medicine, tissue engineering or filtration. Polyamide materials can be formed with various types of additives, extending the application possibilities of the obtained products [10,11,12,13,14,15,16,17,18].

The electrospun fibres feature some advantages, such as a good connection of pores, high specific area and the ability to combine active ingredients at a nanoscale. Several factors, such as solution concentration, solvent type, applied voltage, flow rate and distance between the syringe tip and the collector, play a significant role in the morphological characteristics of the product [19,20,21].

Polyamides are also modified by adding various substances, including micro- and nano-additives. As a result, the functional properties of the obtained materials are modified. For example, polyamide 6 was modified with additives such as graphene [22,23], hydroxyapatite [24], carbon fibre [25], carbon nanotubes [26], biocarbon, nanoclay and many other materials [27]. In the literature, we can find examples of the modification of polyamides aimed at obtaining bioactive materials. According to the literature, many researchers have obtained polyamide materials that are both active against micro-organisms and have the properties that support the treatment process. Polyamide 6 was modified with propolis [28], ZnO [29], copper/boic acid [30], TiO_2_ [31] and silver particles [32].

Silver (AgNPs) and gold (AuNPs) nanoparticles are used to obtain bioactive materials. Depending on the reduction conditions or reducing compounds, we obtain metal nanoparticles of various sizes and shapes [33,34]. Copper has antibacterial properties and is much cheaper than silver nanoparticles. The work attempts to obtain bioactive polyamide fibres (PA6) with the addition of acetanilide and copper sulphate pentahydrate [35]. Copper sulphate is a type of salt that occurs as a series of compounds which differ in their degree of hydration. The anhydrous form is a pale green or grey–white powder, while the pentahydrate (CuSO_4_ · 5H_2_O) used as an additive to obtain polyamide 6 bioactive fibres is light blue in colour.

In these studies, new fibres based on polyamide 6/acetanilide/copper sulphate were obtained. Copper sulphate is reduced to copper microparticles (CuMPs) during fibre formation. The amount of the additive used affects the colour of the fibres obtained. Studies were carried out on the effect of the number of additives used on the morphological and structural properties, and above all, on the antibacterial properties.

Structural changes under the influence of the additives are the subject of spectroscopic and X-ray diffraction studies, which also concern the additions to the polyamide 6 matrix proposed in the tests.

The crystal structure of polyamides is dominated by hydrogen bonds. Polyamide 6 can crystallize into α and γ forms, although the α form is predominant. The two structures are interchangeable in such a way that the α form can be changed into the γ form by means of treatment in an aqueous iodine and potassium iodide solution, and the γ form can be transformed into the α form through treatment with an aqueous phenol solution [36,37,38,39,40].

This article describes a method of obtaining polyamide fibres with additives that has not been described in the literature. The additives used and the process of their formation result in polyamide fibres with bioactive properties. Positive results were obtained for the selected representatives of Gram-positive and Gram-negative bacterial strains, which were observed under a microscope, where zones of inhibition of their growth were visible. The influence of the number of additives used on the morphological structure of new fibres was analysed using optical and scanning electron microscopy. The influence of the number of additives used on the molecular structure was analysed using spectroscopic and X-ray methods.

## 2. Materials and Methods

### 2.1. Materials

The polyamide 6 (PA6) commercial-grade natural Tarnamid^®^ T-27 is produced by Grupa Azoty S.A. (Tarnów, Poland) and features the following specifications: density, 1.14 g/cm^3^; melt flow index MFI of 44.3 g/10 min (230 °C, 2.16 kg); melting point, 221 °C; strength at yield, 63.6 MPa; elongation at yield, 14.5 %; 1 Young’s modulus, 2367 MPa; Charpy notched impact strength, (23 °C) 2.33 kJ/m^2^; and Vicat thermal resistance, 192 °C.

Acetanilide (C_8_H_9_NO) and copper sulphate pentahydrate (CuSO_4_ · 5H_2_O) were supplied by Avantor Performance Materials Poland S.A. (Gliwice, Poland) and used directly without further purification.

### 2.2. Fibre Formation

**Figure 1:**
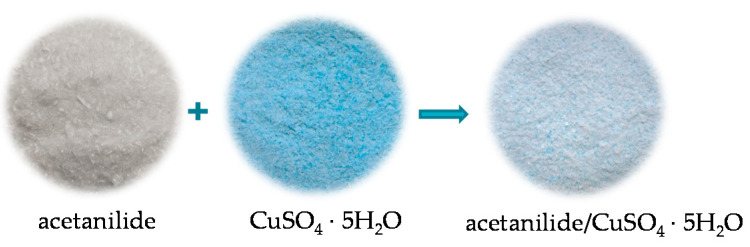
Scheme of combining the components of the mixture.

In the first stage of fibre formation, copper (II) sulphate (VI) pentahydrate was ground in a mortar to ensure an even distribution of PA6 granules on the surface. Then, 10 g of acetanilide was added to the ground copper sulphate and ground again. A particulate solid mixture of copper (II) sulphate pentahydrate with acetanilide was obtained. In order to evenly distribute the mixture in the granulate, 10 g of acetone was added, which partially dissolved the acetanilide. This made it possible to cover the PA6 granules with the modifying mixture, as shown in the diagram in Figure 1 below.

**Figure 2:**
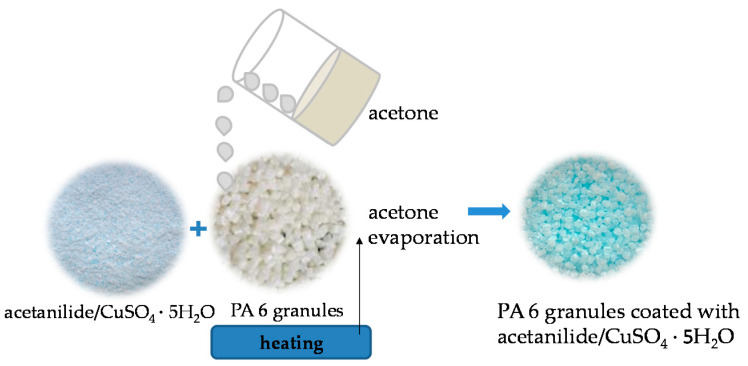
Scheme of mixing additives with polyamide 6.

The next step was to combine the mixture of additives with the appropriate number of polyamide 6 granules, depending on the concentration of modifiers used. Forming fibres with additives requires the proper dispersion of modifiers in the polymer matrix. For this purpose, 5 g of acetone was added to the prepared mixture of modifiers, which was followed by polyamide 6 granules. Then, the polymer and additives were mixed and heated to evaporate the acetone. As a result, PA6 granulate coated with a mixture of acetanilide and copper sulphate pentahydrate was obtained, as shown is the diagram in Figure 2 below.

The fibres were formed using a Zamak Mercator (Skawina, Poland) twin screw extruder. The mixture was placed in the extruder hopper. The fibres were formed using a single screw extruder at 225 °C. A single-hole spinneret with a diameter of 0.5 mm was used. The fibres stretch ratio was 200%, and the rate of fibres collection was 500 m/min. Table 1 shows the composition of the different blends.

### 2.3. Methods of Materials Characterization

Scanning electron microscopy (SEM) was used to observe the structure of the samples, which were characterized by JSM 5500 LV made by JEOL (Tokyo, Japan). The microscope was operated in back-scattered electron mode, using an accelerating voltage of 10 kV. The samples were gold coated in a Jeol JFC 1200 (Tokyo, Japan) ion sputter coater.

The surfaces of the fibres were observed by an optical microscope (Reichert, Viena, Austria) equipped with an ARTCAM CCD camera (Olympus, Tokyo, Japan) controlled by the Motic Images Plus 2.0 computer program. The images were taken using the transmission method in the light passing through the sample.

The change in the chemical composition of fibres during exposure in the soil was analysed by Fourier transform infrared spectroscopy (FTIR). The spectroscopic investigations were carried out using a Nicolet 6700 Fourier Transform spectrophotometer (Thermo Scientific, Waltham, MA, USA) with OMNIC 9.0 software, and an MTEC model 300 photoacoustic accessory (Thermo Scientific, Waltham, MA, USA) was used in the FTIR spectroscopic analysis. The spectral region was as follows: 4000–500 cm^−1^, resolution: 4 cm^−1^, number of scans: 64 of the solid samples. Each spectrum was analysed with the use of a linear baseline and pre-processed by means of Fourier smoothing.

The spectrometer MAGNA-IR 860 (Thermo Scientific, Waltham, MA, USA) and NICOLET with an FT-Raman accessory were used to record the Raman spectra of the samples. The solid samples were then irradiated with a 1064 nm line YAG laser, and scattered radiation was collected with 8 cm^−1^ resolution.

Wide-angle X-ray scattering (WAXS) studies were performed using a URD-65 Seifert diffractometer (Rich. Seifert & Co. Röntgenwertk, Ahrensburg, Germany) employing the Bragg–Brentano reflection geometry method. CuKα radiation (λ = 1.54 Å) was emitted at an accelerating voltage of 40 kV and an anode current of 30 mA. The monochromatization of the radiation beam was achieved using a graphite monochromatizer. A scintillation counter was used as a detector. The tests were carried out in the range of 2θ from 3° to 60° in steps of 0.01°.

The small-angle X-ray scattering (SAXS) investigations were performed using an MBraun camera that utilized a conventional Kratky collimation system (HECUS-MBraun Graz X-ray Systems, Graz, Austria). The front of the camera was directly mounted on top of the tube shield of a stabilized Philips PW 1830 X-ray generator. The X-ray tube was operated at a power of 1.5 kW. CuKα radiation was used. Scattered radiation was recorded in an acquisition time of 1200 s, using an MBraun linear position-sensitive detector, model PSD 50. The detector had 1024 channels with a channel-to-channel distance of 52 μm. Analysis of the SAXS data was carried out using a normalized one-dimensional correlation function.

The elemental chemical analysis was performed using a Phenom ProX microscope (AM Eindhoven, The Netherlands) with a fully integrated EDS detector and software (v. 2.9.0, Eindhoven, The Netherlands).. The distribution of the different elements in the fibres was evaluated with the element identification (EID) software (v. 2.9.0, Eindhoven, The Netherlands). package and a specially designed and fully integrated energy-dispersive spectrometer (EDS).

### 2.4. Microbiological Activity

The materials were exposed to the Gram-positive *Staphylococcus aureus* ATCC No. 25923, Gram-negative *Escherichia coli* ATCC No. 25922, and *Pseudomonas aeruginosa* ATCC No. 27853. The microorganisms were purchased from ATCC. The following media were used for the cultivation of microorganisms: Chapman agar, Mac Conkey agar, Cetrymide agar. Microorganisms were incubated at 37 °C for 24 h. The sterile physiological salt (2 cm^3^) was poured into the cultured bacteria. Grown cultures were washed out with 1 mL of saline solution and added to the sterile selective agar. The compressed fibre samples were applied to inoculated agars. Samples were incubated at 37 °C, for 24–48 h. The assessment of antibacterial activity consisted of placing a sample on an agar substrate containing bacterial culture and observing its growth under and around the sample. The zones of inhibition of microbial growth observed in the studies were determined using an optical microscope.

## 3. Results

**Figure 3:**
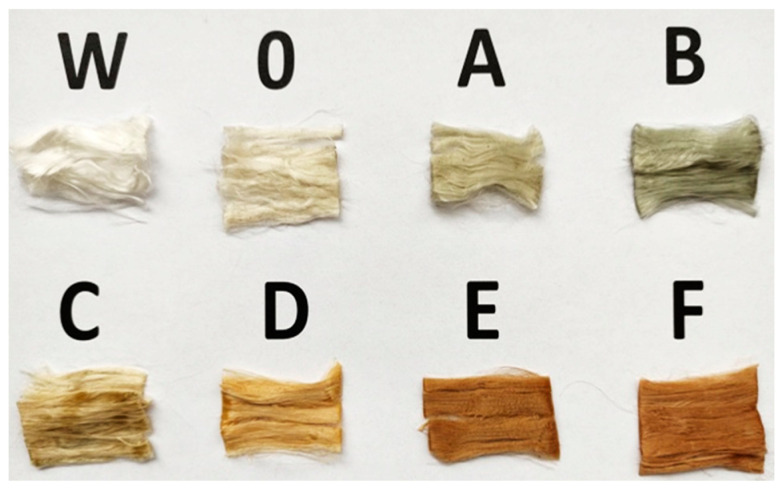
Digital image of PA6 fibres with additives.

As a result of the extrusion of the pre-milling melt, polyamide 6 fibres (sample W) and PA6 fibres with 10 % acetanilide (sample 0) were obtained. Precipitation of acetanilide in the polyamide fibres occurs after the polyamide solidifies, which results in internal optical density differences. As a result, the shade and gloss of the fibres is changed. Fibres with acetanilide (sample 0) are creamier and less shiny compared to fibres with PA6 alone (sample W). PA6 fibres with the addition of 10% acetanilide and various CuSO_4_ · 5H_2_O content from 0.196 to 5.894 g (samples A, B, C, D, E, F) no longer have the colour of copper sulphate pentahydrate as in the granulate before melting. As a result of the reduction of copper ions during fibre formation and the addition of acetanilide, the colour of the obtained fibres changed from grey—sample A with the content of 0.05 g Cu—to brown—sample F with the content of 1.5 g Cu. The estimated content of reduced copper in the form of microparticles in the obtained fibres is presented in Table 1. Below is an image of all the obtained fibres taken from the coils, which shows that the addition of CuSO_4_ · 5H_2_O to polyamide 6 affects the colour of the obtained fibres (Figure 3).

The addition of acetanilide to polyamide fibres is intended to create channels inside the fibre structure that penetrate deep into the fibres. Acetanilide is soluble in water at 0.696 g/100 g; it also sublimes at ambient temperature. The channels that have been set up allow better wetting of the fibres in the aqueous environment and enable reducing the copper salt to CuMPs and their migration to the fibre surface. The lack of acetanilide in the fibres may cause CuMPs to be encapsulated in the polymer matrix, thus preventing ion migration to the fibre surface. The release of Cu ions on the surface of the fibres should result in better bioactivity of the fibres. This is the main purpose of forming such modified polyamide fibres.

**Figure 4:**
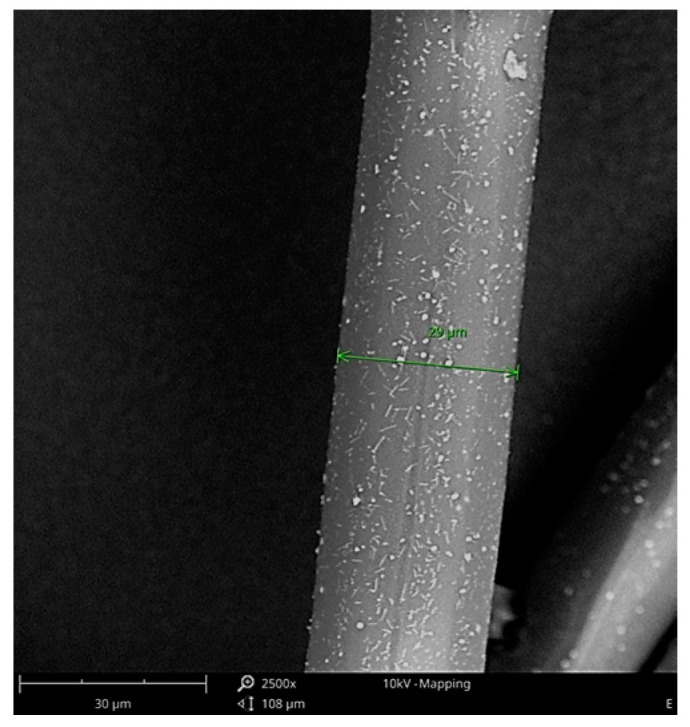
SEM photograph of the fibre with the highest content (1.5%) of CuMPs (sample F).

Figure 4 shows an SEM photograph for fibres with the highest content (1.5%) of CuMPs, i.e., sample F. In the photograph, the diameter of the example fibre is 29 µm.

**Figure 5:**
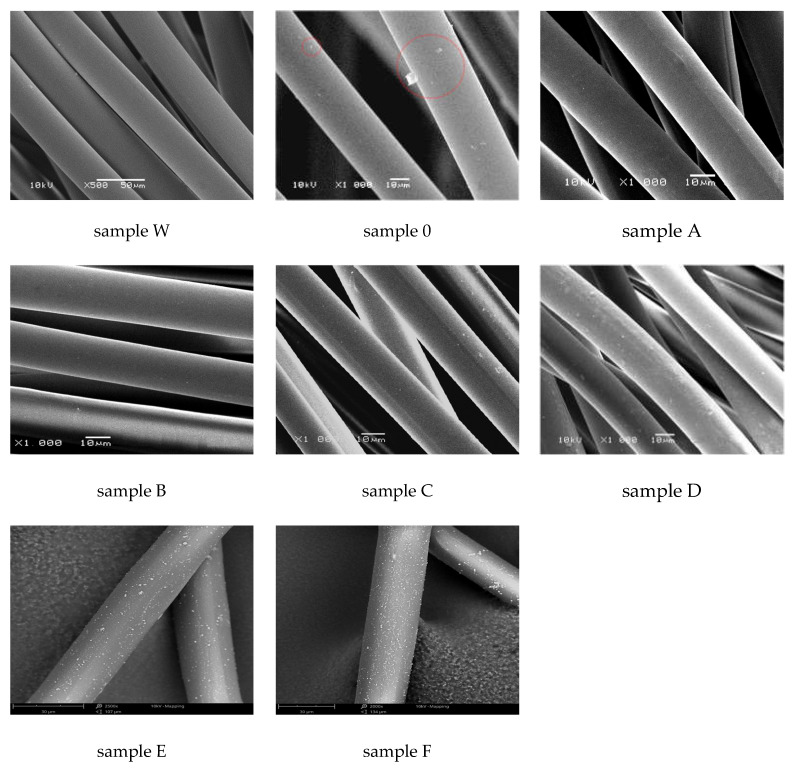
SEM photographs of the surface of fibres with various concentrations of CuMPs. Acetanilide crystals are marked with red circles.

Scanning electron microscopy (SEM) enabled the observation of the surface morphology of the obtained fibres (Figure 5).

SEM photographs for PA6 fibres without additives (samples W) show that the fibres are characterized by a smooth surface. The addition of 10% acetanilide (sample 0) causes the appearance of acetanilide crystals on the surface of the fibres (red circles), which are precipitated by diffusion to the surface and resublimation. SEM photographs of the surface of PA6 fibres with increasing content of CuMPs (samples A, B, C, D, E, F) show the effect of the amount of additive on the morphological structure of the fibre surface. For these fibres, we observe an increasing number of inclusions on the fibre surface with the number of copper sulphate addition and further reduction to CuMPs, which occur evenly on the surface of the fibres.

**Figure 6:**
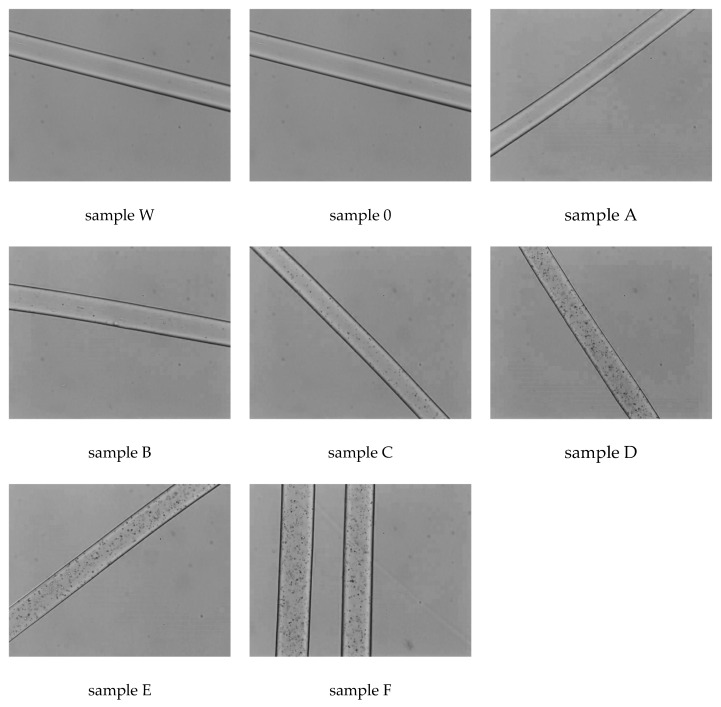
The optical microscopy photographs of all fibres with various concentrations of CuMPs.

The examination of the surface morphology for the fibres was carried out by optical microscopy (Figure 6). Photographs of all obtained fibres were taken in transmission light in oil immersion.

In the optical microscopic photographs for PA6 fibres without additives (sample W) and for polyamide fibres with the addition of 10% acetanilide (sample 0), we do not observe the influence of the additive on the morphological structure of polyamide fibres. The same is true for fibres with the lowest amount of CuSO_4_ · 5H_2_O (sample A). This may be due to the small number of CuMPs that do not diffuse visible light in a noticeable way in photographs. Dark inclusions appear in the photographs obtained for fibres with a 0.1% addition of CuMPs (sample B). With a higher content of CuMPs, we observe more dark inclusions in transmitted light, which are evenly distributed along the length of the fibres.

**Figure 7:**
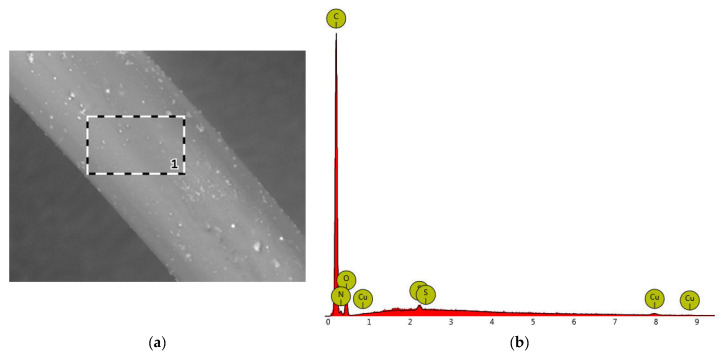
(**a**) SEM photographs with a marked area of mapping on the surface of sample fibres (the dashed line shows the scanning path of the EDS probe); (**b**) EDS analysis of particles.

EDS tests were performed for samples from the sample “E”. EDS spectra for the elemental analysis were obtained by positioning the laser beam in the middle of randomly selected particles, as shown in Figure 7a. A representative EDX spectrum is shown in Figure 7b.

The main elements identified in the particles were C, O, N, Cu and S (Table 2).

The results of the EDS tests for sample E (1.0 g Cu) indicate a lower content of CuMPs in the selected area on the surface of the tested fibres that was accessible to the electron beam. This may be due to the different distribution of CuMPs both on the surface and inside the polymer matrix. An additional benefit is obtaining a porous structure of the fibres, which will enable the migration of copper ions from the core to the surface of the fibres, therefore having a positive effect on the microbiological activity of the fibres. The addition of acetanilide, which dissolves in water and sublimes from the fibre surface, will result in a better migration of CuMPs.

The interaction of the PA6, acetanilide, CuSO_4_ · 5H_2_O was investigated using FTIR analysis. Some feature wavenumbers of PA6, acetanilide, CuSO_4_ · 5H_2_O are labelled in Table 3.

**Figure 8:**
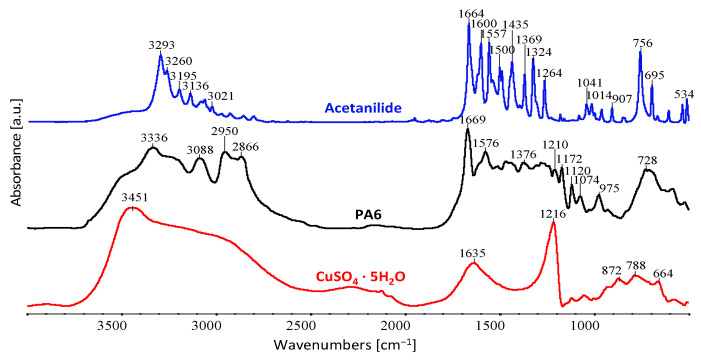
FTIR spectra of fibre-forming components used: acetanilide, PA6, CuSO_4_ · 5H_2_O.

The spectra of all the components used in forming the fibres are shown in Figure 8.

Figure 8 shows the FTIR spectrum of acetanilide, PA6, and CuSO_4_ · 5H_2_O with the characteristic oscillation bands indicated.

The stretching vibration bands of amide I (C=O) and II (C–N) of PA6 are located at 1669 and 1576 cm^−1^ on FTIR spectra, respectively (Figure 8 and Table 3). Peaks of 1210 cm^−1^ were ascribed to the N–H bending vibration of amide III. The absorptions of 2866 cm^−1^ and 2950 cm^−1^ were attributed to the saturated asymmetric and symmetric stretching of C–H. The absorption bands of PA6 at 3336 and 3088 cm^−1^ were assigned to N–H stretching and N–H bending vibration.

The acetanilide spectrum features characteristic peaks at the band values of 3293, 3260, 3195, 3136, 3021, 1664, 1600, 1557, 1435, 1369, 1324, 1264, 1041, 1014, 907, 756, 695 and 534 cm^−1^.

In the CuSO_4_ · 5H_2_O spectrum, the characteristic peaks are located at the band values of 3451, 1635, 1216, 872, 788 and 664 cm^−1^. In these spectra, the peaks over 3000 cm^−1^ can be explained by the structure of crystalline water. The peaks at lower band values can be explained by vibrations between O and non-metal atoms [42].

**Figure 9:**
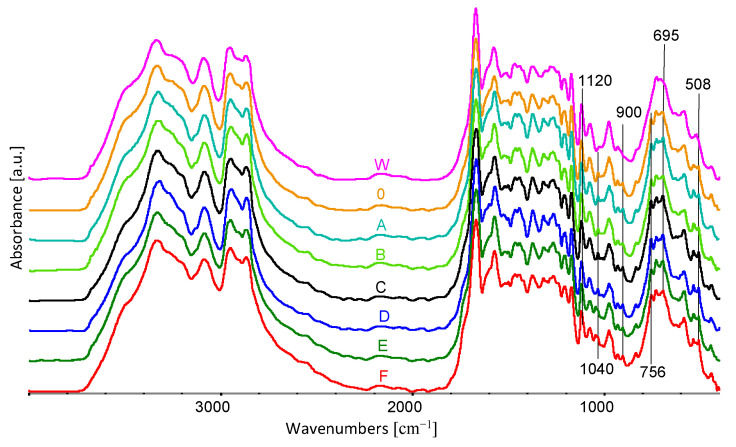
FTIR spectra within a range of 4000–400 cm^−1^ for the fibres.

**Figure 10:**
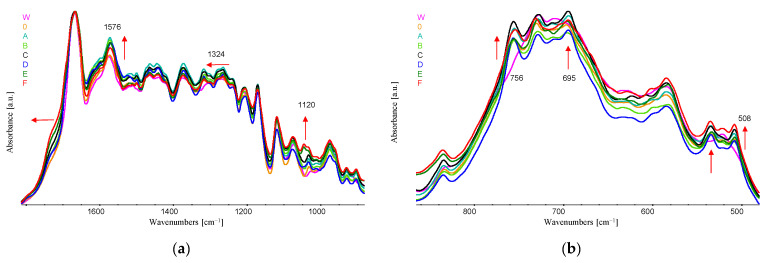
FTIR spectra of all fibres; (**a**,**b**) in a different ranges of wavenumbers. Band shifts are marked with red arrows.

In the spectra (Figure 9 and Figure 10a,b) obtained for fibre samples, we do not observe a significant effect of the 10% addition of acetanilide on the intensity of the bands characteristic for this chemical compound. Slight differences are observed for the wavenumbers at the following positions: 900, 756, 695 and 508 cm^−1^. This is probably related to the strong intermolecular interactions between the amide groups of PA6 and acetanilide. The strong intermolecular interactions are confirmed by the fact that PA6 dissolves in liquid acetanilide.

**Figure 11:**
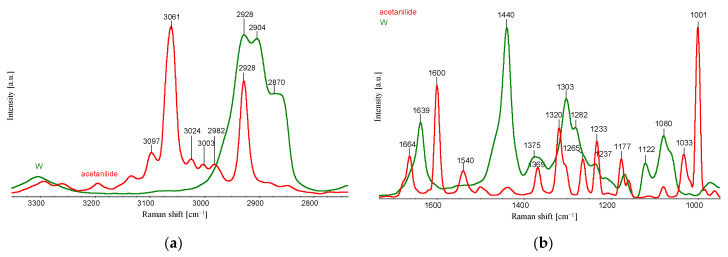
Raman spectra of PA6 (sample W) and acetanilide (**a**,**b**) in different ranges of wavenumbers.

In the spectra of samples with the addition of copper (II) sulphate (VI) pentahydrate (Figure 11a), a slight effect of oxidation on the intensity of the bands is observed in the ranges characteristic for chemical groups containing —O—H bonds at approx. 1640 cm^−1^ and C—O for wavenumbers at maximum positions of 1120 and 1040 cm^−1^. Slight changes also occur in the amide II band at the maximum position of 1576 cm^−1^, which may be caused by partial degradation of the polymer chain.

In the range of skeletal vibrations below 800 cm^−1^ (Figure 10b), we can notice the formation of several bands after adding acetanilide (marked with arrows), which may result from its influence on the conformation of PA6 chains after solidification of the fibres.

The effects observed in the spectra confirm the decomposition of copper (II) sulphate (VI) pentahydrate and its reduction to CuMPs during the fibre formation process. Metallic copper inclusions in the form of CuMPs formed on the fibres do not absorb mid-infrared radiation; therefore, we do not observe changes in the FTIR spectra.

Figure 11 shows the Raman spectra of acetanilide, PA6 with the characteristic oscillation bands indicated.

Figure 11 shows the characteristic maxima for individual bands for the corresponding wavenumbers. The obtained Raman spectra for PA6 and acetanilide are fundamentally different. In the absence of intermolecular interactions, the spectra of the compounds mixture used should contain characteristic bands for individual components.

**Figure 12:**
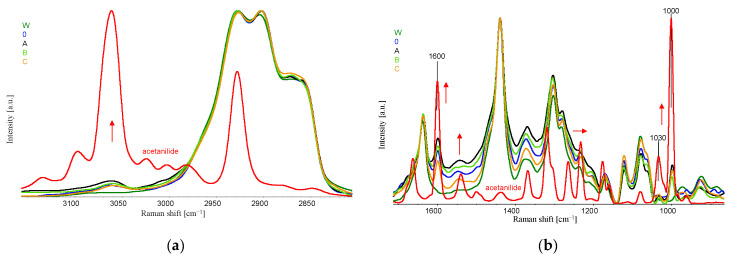
Selected Raman spectra for the obtained fibres and acetanilide (**a**,**b**) in a different ranges of wavenumbers. Band shifts are marked with red arrows.

Strong intermolecular interactions between the polyamide 6 matrix and acetanilide cause a significant reduction in the intensity of the acetanilide bands in the mixture (Figure 12). 

Comparisons of the Raman shift spectra for PA6 fibre samples (samples W) and samples with 10% acetanilide content (sample 0) indicate that the addition of acetanilide results in the appearance of the characteristic band at the maximum wavenumber position of 1600 cm^−1^. A thorough analysis of the spectra with the addition of CuMPs, due to the specific emission of diffuse radiation, does not indicate significant oxidation effects of the polyamide matrix. The strong scattering of the laser beam by the CuMPs microparticles in samples D, E, F prevents the registration of the appropriate Raman spectra.

Because PA6 is a semi-crystalline polymer, the properties of the produced fibres depend on the content of the crystalline phase, which is characterized by the so-called degree of crystallinity (XC). This parameter can be determined using various methods; in the presented paper, it was determined based on the analysis of WAXS diffraction patterns. In order to determine the degree of crystallinity, the WAXS patterns were decomposed into a component resulting from scattering in crystalline regions and a component resulting from scattering in amorphous regions. The deconvolution of scattering curves requires the use of appropriate mathematical functions that theoretically describe the shape of scattering from ordered and disordered regions in the polymer. This work used a linear combination of Gaussian and Cauchy functions, which allowed obtaining a theoretical WAXS curve for a given sample. The entire procedure of decomposing the curves into components and matching the theoretical curve to the experimental one was performed using the WaxsFit computer software (v.2018) [43]. After fitting, the crystallinity index was determined as follows:

(1) $$X_{C}=\frac{A_{\alpha }+A_{\beta }}{A_{\alpha }+A_{\beta }+A_{A}}$$

where

*A_α_* is the sum of integrated areas under the crystalline peaks *α* 200 and *α* 002/202;
*A_β_* is the areas under *β* phase peaks; and
*A_A_* is the integrated area of the amorphous peak.

Parameters *X_α_* and *X_β_* were calculated as a ratio of the *A_α_* or *A_β_*, respectively, to the total area of the scattering curve. Hence, *X_C_* = *X_α_* + *X_β_*.

The crystalline structure of PA6 is complex due to the hydrogen (H) bonds that are established within specific crystallographic planes. PA6 exhibits three crystalline forms that can coexist in various amounts, depending on processing conditions. The structure of the *α*-form is characterized by X-ray diffraction patterns showing two reflections corresponding to *α* 002/202 crystal planes. The structure of the crystalline regions in PA6 is determined by hydrogen bonds, which determine that PA6 exists in two thermodynamically stable crystallographic forms, *α* and *γ*, and in the unstable *β* form. These phases can coexist in various proportions, and the parameters of fibre formation have a decisive influence on this. The method for obtaining the degree of crystallinity described above can also be used to determine the content of individual crystallographic forms. In the present paper, the contents of *α* and *β* phases (*X_α_* and *X_β_*, respectively) were calculated as a ratio of the *A_α_* or *A_β_*, respectively, to the total area of the WAXS diffraction pattern. Hence, *XC* = *A_α_* + *A_β_*. From an experimental point of view, it is difficult to distinguish between *γ* and *β* forms, since both are characterized by X-ray reflections at 2θ ≈ 21°.

**Figure 13:**
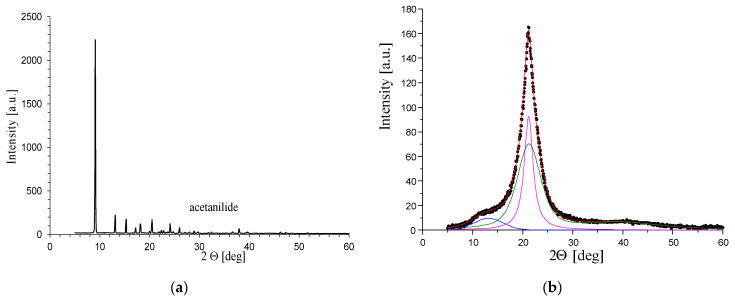

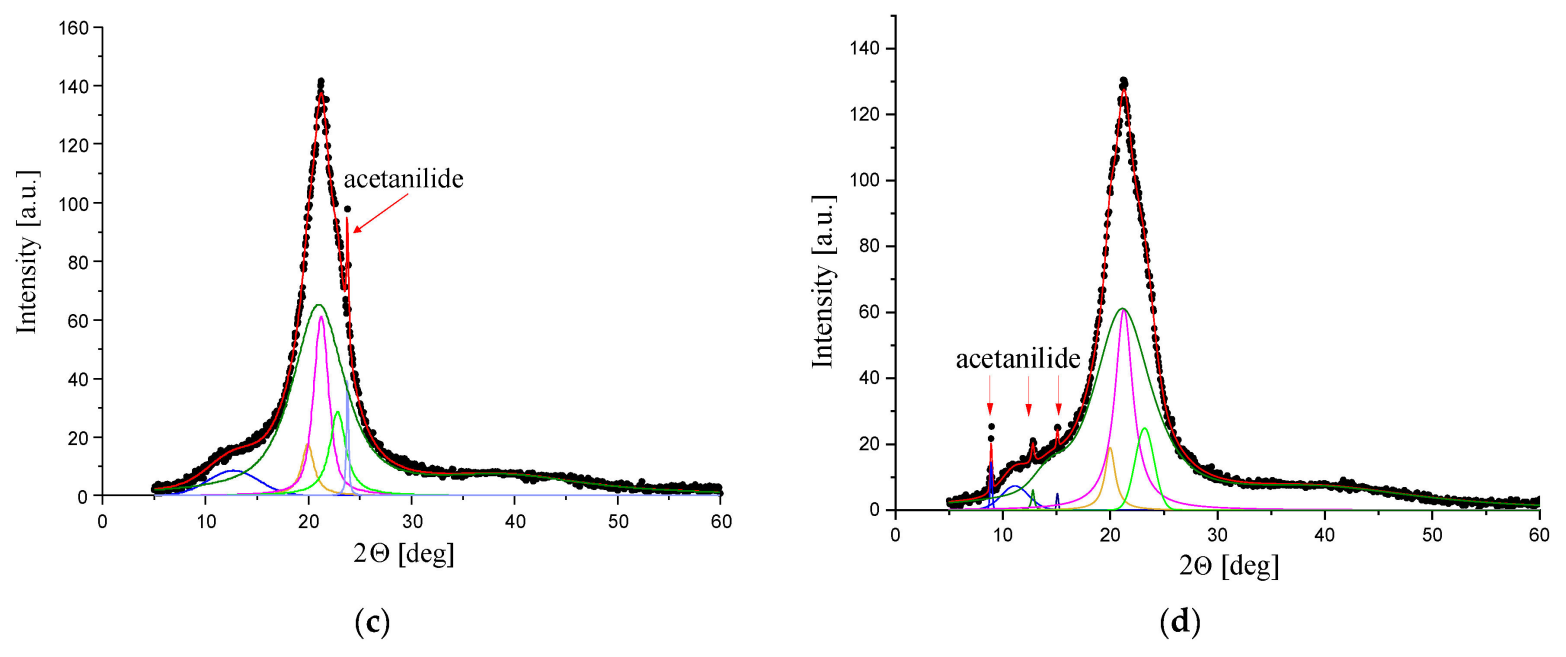
WAXS patterns of acetanilide and fibres: (**a**) acetanilide; (**b**) sample W; (**c**) sample 0; (**d**) sample A.

Figure 13a shows the WAXS curve for pure acetanilide. Figure 13b shows the WAXS curve for pure PA6 fibres obtained under the same forming conditions as fibres containing additives. The diffraction pattern is dominated by the interference maxima characteristic of the mesomorphic *β*-form. The production of PA6 fibres has a tendency to promote the formation of the *β* phase because the crystallization process that takes place in rapid cooling of PA6 from the melt seems to have the effect of freezing the material structure in a transitional state of order.

In order to determine the effect of additives on the molecular structure of the polyamide matrix in composite fibres, WAXS measurements were performed separately for fibres containing only acetanilide and fibres containing various concentrations of CuMPs. The WAXS pattern obtained for fibres containing only acetanilide is shown in Figure 13b. On this curve, two varieties can be observed on the slope of the dominant peak of the *β* phase. The angular positions of these varieties correspond to the positions of the α phase reflections associated with *α* 200 and *α* 002/202 crystal planes

The diffraction patterns of the CuMPs containing fibres are similar to the curve in Figure 13c,d. They are dominated by the *β* phase peak, which is overlapped by the *α* phase reflections (Figure 13c).

**Figure 14:**
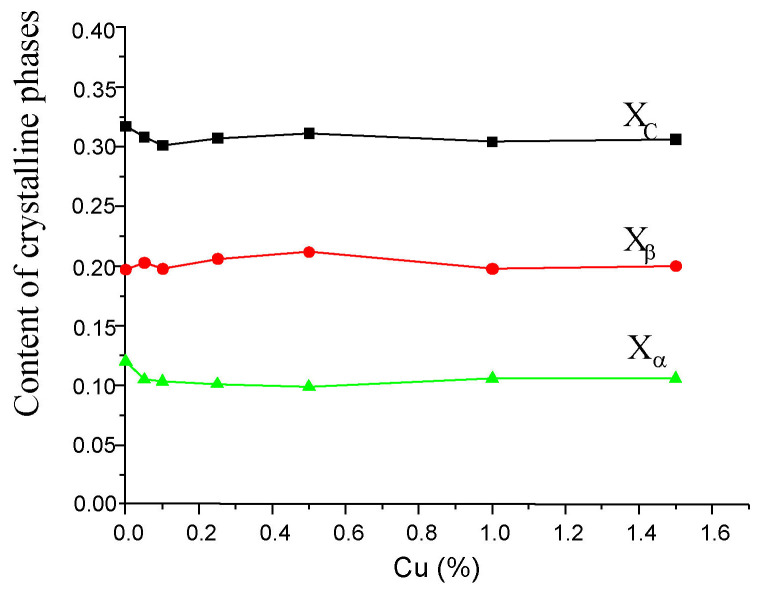
The amount of the crystalline phase depending on the concentration of the additives in the fibres.

The calculated crystallinity index of pure PA6 fibres was found to be 0.362, whereas for modified fibres, the value of this parameter is lower and oscillates around 0.3 regardless of the CuMPs content (Figure 14). In pure PA6 fibres, only the metastable *β* phase is an ordered phase. In composite fibres, some of the PA6 macromolecules arrange themselves to form a stable *α* crystalline phase. However, the α phase content (*X_α_* ≈ 0.1) is twice as low as the *β* phase content (*X_β_* ≈ 0.2).

**Figure 15:**
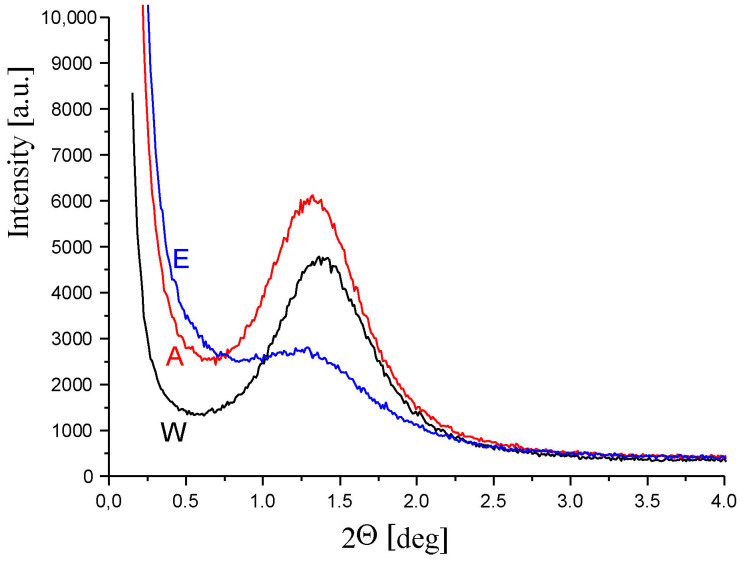
SAXS patterns for a few selected fibres.

Small-angle scattering (SAXS) curves along the fibre axis for a few selected samples are shown in Figure 15. The interference maximum visible on these curves comes from the lamellar structure of the polyamide matrix.

With the increase in the CuMPs content in the fibres, the angular position of this maximum shifts towards smaller scattering angles. This means that the value of the long period of the lamellar structure increases. For fibres presented in Figure 15, the values of the long period are equal to 6.3 nm (sample W), 6.7 nm (sample A) and 7.2 nm (sample E), respectively.

**Figure 16:**
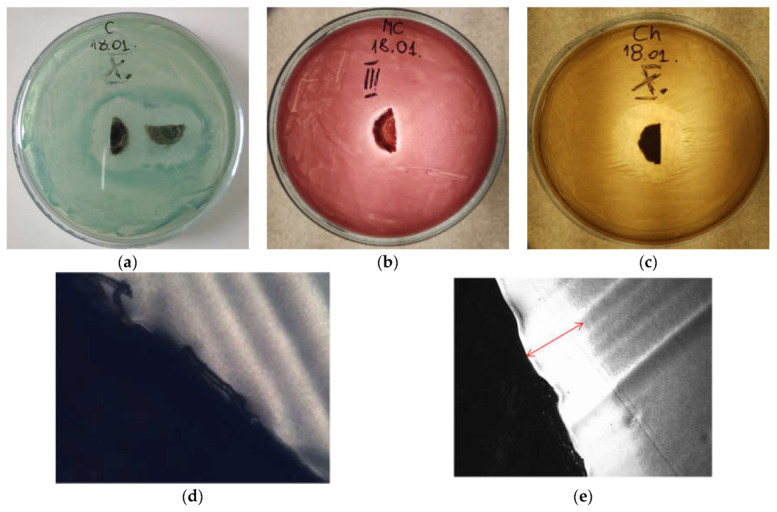
Representative images of the agar plates showing the diameter of the inhibition zone for (**a**) *P. aeruginosa*; (**b**) *E. coli*; (**c**) *S. aureus*; (**d**) sample W—no growth zone of inhibition; (**e**) sample F—with growth zone of inhibition marked. The images were taken after 24 h of bacterial incubation.

The antimicrobial activities of all fibres were tested against Gram-negative bacteria of *Escherichia coli* (*E.coli*), *Pseudomonas aeruginosa* (*P. aeruginosa*), and Gram-positive bacteria of *Staphylococcus aureus* (*S. aureus*). Examples of zones of growth inhibition for the tested bacteria are presented in Figure 16 below.

**Figure 17:**
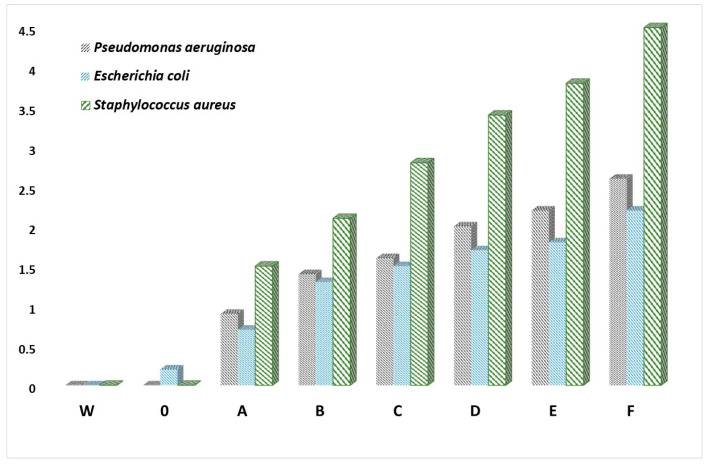
Zones of inhibition of all fibres against *P. aeruginosa*, *E. coli* and *S. aureus*.

The images were taken using an optical microscope. The control samples, which were polyamide 6 fibres (samples W) without additives, did not show bioactivity against the microorganisms used, which is shown in the example in Figure 16d, where we do not observe the zone of microorganism growth inhibition. In Figure 16e, the zone of microbial growth inhibition for the selected sample is marked. In the performed tests, a significant effect of the applied additives on the multiplication or growth inhibition was found for both Gram-negative bacteria *P. aeruginosa* or *E. coli* as well as Gram-positive *S. aureus*. Mean values of growth inhibition zones for all microorganisms for all fibres were also calculated. The results are presented in Figure 17.

The obtained zones of inhibition for Gram-negative *P. aeruginosa* range from ~0.9 to ~2.6 mm, and they increase with the higher concentration of CuMPs in the fibres. The test results obtained for Gram-negative *E. coli* bacteria were similar, although the obtained zones of inhibition ranged from ~0.7 to ~2.2 mm. On the other hand, the higher microbiological activity of the tested fibres was found for Gram-positive *S. aureus* bacteria. The zones of inhibition of bacterial growth and multiplication obtained in this case ranged from ~1.5 to ~4.5 mm. It can be concluded that the inhibition of the Gram-positive *S. aureus* proliferation is more intense and increases with the higher concentration of CuMPs in the fibres.

## 4. Conclusions

Polyamide 6 fibres modified with the addition of acetanilide and copper sulphate pentahydrate were obtained. During fibre formation, copper sulphate was reduced to copper microparticles as a result of high temperature. An observable effect of this process was a change in the colour of the obtained fibres. The fibres are characterized by microbiological activity against bacteria, both Gram-positive *S. aureus* and Gram-negative *P. aeruginosa* and *E. coli*. These fibres show a higher microbiological activity against Gram-positive bacteria *S. aureus*. The microbiological activity of fibres increases with the addition of CuMPs, but this effect is not directly proportional to the content of CuMPs in the fibres. The probable cause of this phenomenon is the formation of large agglomerates of CuMPs on the fibre surface. The migration of copper ions may be affected by the porosity of the fibres, which, in this case, is conditioned by the same content of acetanilide as in a pouring agent.

## Figures and Tables

**Table 1:** Compositions of the prepared PA6/acetanilide/copper sulphate pentahydrate blends.

The Composition of the Spinning Mixture (g)				
Designations of Samples	PA6	Acetanilide	CuSO_4_ · 5H_2_O	Cu
W	100	-	-	-
0	90	10	-	-
A	89.95	10	0.196	0.05
B	89.90	10	0.393	0.10
C	89.75	10	0.982	0.25
D	89.50	10	1.965	0.50
E	89.00	10	3.929	1.00
F	88.50	10	5.894	1.50

**Table 2:** EDS analysis of sample “E”.

Symbols of Elements	Atomic Percent—Sample E
C	74.78
O	13.14
N	11.07
Cu	0.83
S	0.18

**Table 3:** Wavenumbers of the bands observed in the FTIR spectra of PA6, acetanilide and CuSO_4_ · 5H_2_O bands [41].

Sample	Wavenumber from FTIR, cm^−1^	Oscillation Bands
PA6, acetanilide	3600–3200	N-H, O-H
	3100–2800	C-H
	1680–1640	C=O
	1590–1540	C-N
	1400–1200	C-H
	1250–1000	C-O
	>1000–400	skeletal
CuSO_4_ · 5H_2_O	3600–3200	O-H
	1680–1600	O-H
	1300–1150	1300–1150
	>1000–400	skeletal

## Data Availability

All data are presented in the paper.
